# Phylogenetic history demonstrates two different lineages of dengue type 1 virus in Colombia

**DOI:** 10.1186/1743-422X-7-226

**Published:** 2010-09-14

**Authors:** Jairo A Mendez, Jose A Usme-Ciro, Cristina Domingo, Gloria J Rey, Juan A Sanchez, Antonio Tenorio, Juan C Gallego-Gomez

**Affiliations:** 1 Laboratorio de Virología, Instituto Nacional de Salud, Avenida/Calle 26 No. 51-20, Bogotá D.C.,Colombia; 2 Viral Vector Core and Gene Therapy, Neurosciences Group, Sede de Investigación Universitaria, Universidad de Antioquia, A.A. 1226, Medellín, Colombia; 3 Laboratorio de Arbovirus y Enfermedades Víricas Importadas, Centro Nacional de Microbiología, Instituto de Salud Carlos III, Carretera Majadahonda-Pozuelo Km2, Majadahonda (28220), Madrid, Spain; 4 Departamento de Ciencias Biológicas-Facultad de Ciencias, Laboratorio BIOMMAR, Universidad de los Andes, Carrera 1 No. 18a-10 Bloque J-309, Bogotá D.C.,Colombia; 5 Current address: Robert Koch Institute, Nordufer 20, Berlin 13353, Germany

## Abstract

**Background:**

Dengue Fever is one of the most important viral re-emergent diseases affecting about 50 million people around the world especially in tropical and sub-tropical countries. In Colombia, the virus was first detected in the earliest 70's when the disease became a major public health concern. Since then, all four serotypes of the virus have been reported. Although most of the huge outbreaks reported in this country have involved dengue virus serotype 1 (DENV-1), there are not studies about its origin, genetic diversity and distribution.

**Results:**

We used 224 bp corresponding to the carboxyl terminus of envelope (E) gene from 74 Colombian isolates in order to reconstruct phylogenetic relationships and to estimate time divergences. Analyzed DENV-1 Colombian isolates belonged to the formerly defined genotype V. Only one virus isolate was clasified in the genotype I, likely representing a sole introduction that did not spread. The oldest strains were closely related to those detected for the first time in America in 1977 from the Caribbean and were detected for two years until their disappearance about six years later. Around 1987, a split up generated 2 lineages that have been evolving separately, although not major aminoacid changes in the analyzed region were found.

**Conclusion:**

DENV-1 has been circulating since 1978 in Colombia. Yet, the phylogenetic relationships between strains isolated along the covered period of time suggests that viral strains detected in some years, although belonging to the same genotype V, have different recent origins corresponding to multiple re-introduction events of viral strains that were circulating in neighbor countries. Viral strains used in the present study did not form a monophyletic group, which is evidence of a polyphyletic origin. We report the rapid spread patterns and high evolution rate of the different DENV-1 lineages.

## Background

Dengue virus infection has been an important impact on humans over the last several years, with an estimated 50 million dengue infections and an average of 1 million cases reported annually in more than 100 countries in tropical and subtropical regions [1-5]. This mosquito-borne flavivirus causes a wide spectrum of clinical manifestations in humans, which include an acute self-limited flu-like illness known as dengue fever (DF). DF is characterized by headache, myalgia, arthralgia, retro-orbital pain and sometimes maculopapular rash. Dengue haemorrhagic fever (DHF) is a severe illness documented by haemoconcentration (haematocrit increase by 20%) and evidence of plasma leakage such as pleural effusion and ascites as the major pathophysiological features. In some patients, DHF may progress to hypovolemic shock (Dengue Shock Syndrome, DSS) with circulatory failure [2,6-8].

Dengue virus (DENV) is an enveloped virus with a positive sense ssRNA of about 11 kb coding a single open reading frame for three structural proteins, core (C), pre-membrane/membrane (prM/M) and envelope (E), and seven non-structural proteins (NS1, NS2a, NS2b, NS3, NS4a, NS4b, NS5). Based on serological analysis, DENV can be differentiated as four distinct serotypes (DENV-1, DENV-2, DENV-3 and DENV-4), each one with the capacity to infect and cause even the more severe manifestation, although some serotypes have been isolated more frequently in DHF epidemics. On the other hand, evolution studies and molecular epidemiology using nucleotide sequences from the DENV genome have demonstrated the occurrence of genotype clades within each serotype [9-28]. For this reason, genetic characterization of DENV has become a critical issue for understanding epidemic patterns of viral spread. At the same time, the important role of DENV itself in disease severity has also been proposed rather than the immune enhancement developed after subsequent infection with heterologous serotypes [1,7,29]. The increase in virus transmission over the last 50 years has possibly increased its adaptive potential. In addition, host factors such as the age, race, presence of non-neutralazing cross-reactive antibodies and possibly chronic diseases could act as selective pressures, resulting in more virulent genotypes that may be associated with DHF/DSS [9,17,29-34].

Four DENV serotypes have been involved in Colombian epidemics, although DENV-1 and DENV-2 have the higher circulation rate since 1971[5,6,21]. Moreover, since the first case of DHF in Colombia at the end of 1989, these two serotypes have been associated with severe disease. To date, DENV-1 falls into five clades designated as genotype I (Southeast Asia, China and East Africa), genotype II (Thailand), genotype III (Malaysia), genotype IV (South Pacific) and genotype V(America, Africa). Additionally, the existence of lineages with distinctive geographical and temporal relationships had been suggested [12,20,26,28,35-38]. Due to the importance of DENV in public health, the particular goals of this research were to reconstruct the phylogenetic history of DENV-1 and to date the phylogenetic tree using isolation time as calibration points to establish date of introduction of virus and rate evolution patterns of virus in Colombia.

## Results

### Virus recovery and confirmation

Seventy four viruses obtained from symptomatic patients were isolated in mosquito cell culture and subsequently identified as DENV-1 serotype by monoclonal antibodies and confirmed by RT-PCR methods. From the 74 samples, it was not possible to obtain the exact geographic origin of 10 samples. The remaining 64 isolates are listed in Table 1 indicating locality, isolation year, accession number and genotype.

**Table 1:** Colombian DENV-1 isolates sequenced and analyzed in the present study

ISOLATE*	LOCALITY	ISOLATION YEAR	GENOTYPE/LINEAGE**	ACCESSION NUMBER
DENV-1/CO/261_Atlantico/1978	Atlántico	1978	V/1	HM067643
DENV-1/CO/150_Choco/1979	Chocó	1979	V/1	HM067617
DENV-1/CO/263_Choco/1979	Chocó	1979	V/1	HM067644
DENV-1/CO/267_Valle/1983	Valle	1983	I	HM067645
DENV-1/CO/188_Guaviare/1987	Guaviare	1987	V/1	HM067618
DENV-1/CO/191_SanAndres/1996	San Andrés	1996	V/2	HM067619
DENV-1/CO/192_Santander/1997	Santander	1997	V/1	HM067620
DENV-1/CO/255_Santander/1997	Santander	1997	V/1	HM067642
DENV-1/CO/589_Casanare/1997	Casanare	1997	V/1	HM067678
DENV-1/CO/98_SanAndres/1998	San Andrés	1998	V/1	HM067615
DENV-1/CO/196_Huila/1998	Huila	1998	V/1	HM067621
DENV-1/CO/197_Santander/1998	Santander	1998	V/1	HM067622
DENV-1/CO/198_Tolima/1998	Tolima	1998	V/1	HM067623
DENV-1/CO/199_Cundinamarca/1998	Cundinamarca	1998	V/1	HM067624
DENV-1/CO/204_Arauca/1998	Arauca	1998	V/2	HM067625
DENV-1/CO/206_SanAndres/1998	San Andrés	1998	V/1	HM067626
DENV-1/CO/207_SanAndres/1998	San Andrés	1998	V/1	HM067627
DENV-1/CO/208_SanAndres/1998	San Andrés	1998	V/1	HM067628
DENV-1/CO/210_SanAndres/1998	San Andrés	1998	V/1	HM067629
DENV-1/CO/211_SanAndres/1998	San Andrés	1998	V/1	HM067630
DENV-1/CO/251_Arauca/1998	Arauca	1998	V/2	HM067638
DENV-1/CO/252_SanAndres/1998	San Andrés	1998	V/1	HM067639
DENV-1/CO/269_Santander/1998	Santander	1998	V/1	HM067646
DENV-1/CO/270_Santander/1997	Santander	1998	V/1	HM067647
DENV-1/CO/280_Cundinamarca/1998	Cundinamarca	1998	V/2	HM067649
DENV-1/CO/319_Arauca/1998	Arauca	1998	V/1	HM067651
DENV-1/CO/498_Casanare/1998	Casanare	1998	V/2	HM067666
DENV-1/CO/501_Bogota/1998	Bogotá	1998	V/2	HM067669
DENV-1/CO/597_Huila/1998	Huila	1998	V/1	HM06768
DENV-1/CO/324_Cundinamarca/1998	Cundinamarca	1998	V/2	HM067652
DENV-1/CO/515_Guaviare/1999	Guaviare	1999	V/2	HM067676
DENV-1/CO/521_Amazonas/1999	Amazonas	1999	V/2	HM067677
DENV-1/CO/213_Caqueta/2000	Caquetá	2000	V/2	HM067631
DENV-1/CO/214_Nariño/2000	Nariño	2000	V/2	HM067632
DENV-1/CO/215_Cesar/2000	Cesar	2000	V/2	HM067633
DENV-1/CO/216_Cachicamo/2000	Guaviare	2000	V/2	HM067634
DENV-1/CO/253_Nariño/2000	Nariño	2000	V/1	HM067640
DENV-1/CO/277_Nariño/2000	Nariño	2000	V/1	HM067648
DENV-1/CO/288_Cachicamo/2000	Guaviare	2000	V/2	HM067650
DENV-1/CO/329_Caqueta/2000	Caquetá	2000	V/2	HM067653
DENV-1/CO/485_Caqueta/2000	Caquetá	2000	V/2	HM067663
DENV-1/CO/499_Guaviare/2000	Guaviare	2000	V/2	HM067667
DENV-1/CO/506_Cesar/2000	Cesar	2000	V/2	HM067671
DENV-1/CO/508_Tolima/2001	Tolima	2001	V/2	HM067672
DENV-1/CO/254_Quindio/2002	Quindío	2002	V/1	HM067641
DENV-1/CO/347_Quindio/2002	Quindío	2002	V/2	HM067654
DENV-1/CO/348_Quindio/2002	Quindío	2002	V/2	HM067655
DENV-1/CO/351_Guajira/2002	Guajira	2002	V/2	HM067656
DENV-1/CO/486_Quindio/2002	Quindío	2002	V/2	HM067664
DENV-1/CO/487_Quindio/2002	Quindío	2002	V/2	HM067665
DENV-1/CO/502_Guaviare/2002	Guaviare	2002	V/2	HM067670
DENV-1/CO/232_Meta/2003	Meta	2003	V/2	HM067635
DENV-1/CO/364_Caqueta/2003	Caquetá	2003	V/2	HM067657
DENV-1/CO/381_Valle/2003	Valle	2003	V/2	HM067658
DENV-1/CO/387_Valle/2003	Valle	2003	V/2	HM067659
DENV-1/CO/123_Putumayo/2004	Putumayo	2004	V/2	HM067616
DENV-1/CO/235_Putumayo/2004	Putumayo	2004	V/2	HM067636
DENV-1/CO/509_Huila/2004	Huila	2004	V/2	HM067673
DENV-1/CO/510_Huila/2004	Huila	2004	V/2	HM067674
DENV-1/CO/511_Huila/2004	Huila	2004	V/2	HM067675
DENV-1/CO/250_Putumayo/2005	Putumayo	2005	V/2	HM067637
DENV-1/CO/446_Putumayo/2005	Putumayo	2005	V/2	HM067660
DENV-1/CO/457_Putumayo/2005	Putumayo	2005	V/2	HM067661
DENV-1/CO/471_Guainia/2005	Guainía	2005	V/2	HM067662

* Internal isolations code, Laboratorio de Virología, Instituto Nacional de Salud

** Putative lineage established in the present study

### Phylogenetic reconstruction of DENV-1

**Figure 1:**
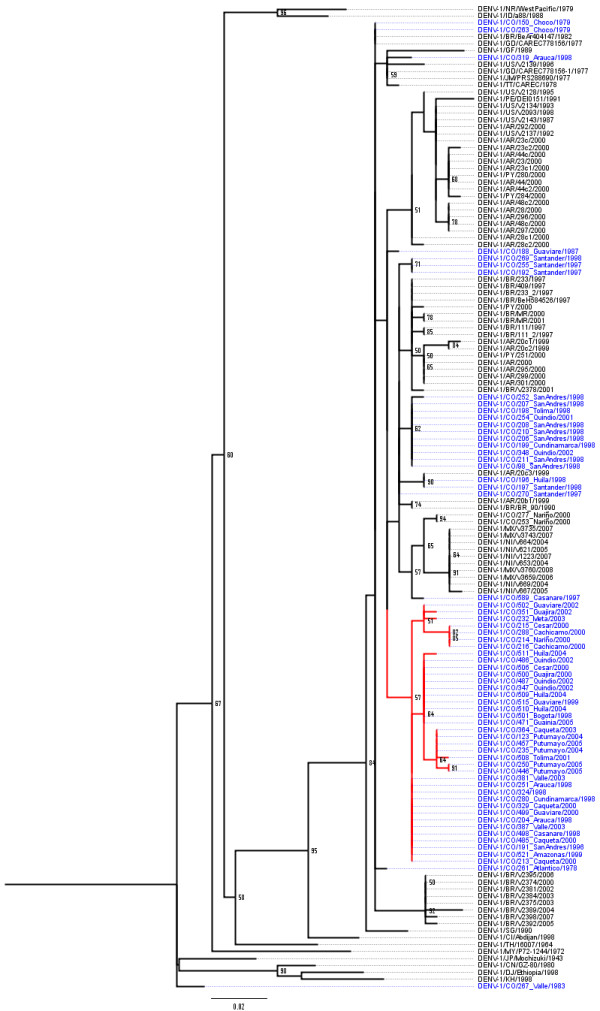
**Evolutionary relationships of DENV-1**. Phylogenetic tree of 126 DENV-1 isolates worldwide. It was computed using the Maximum Likelihood method. The optimal tree with the sum of branch length = 0.596 is shown. The tree is drawn to scale, with branch lengths in the same units as those of the evolutionary distances used to infer the phylogenetic tree. Significant bootstrap values (≥ 50) are indicated. Clade supporting the putative lineage 2 is show in red. Colombian isolates are in blue. There were a total of 221 positions in the final dataset. Phylogenetic analyses were conducted in PAUP*

Sequences from the carboxyl terminus of the envelope (E) gene from the 74 Colombian DENV-1 isolates were aligned in CLUSTAL W [39,40] and compared with 52 previously reported sequences elsewhere, resulting in a trivial alignment as long as there were no indels in the sequences alignment. The Maximum Likelihood analysis comparing 126 sequences is presented in figure 1. Previously reported genotypes were represented in the tree and placed most of the Colombian isolates nesting in the genotype V clade (America, Africa) and were closely related to Argentina, Brazil and Paraguay virus strains. Nevertheless, the oldest sequences

**Figure 2:**
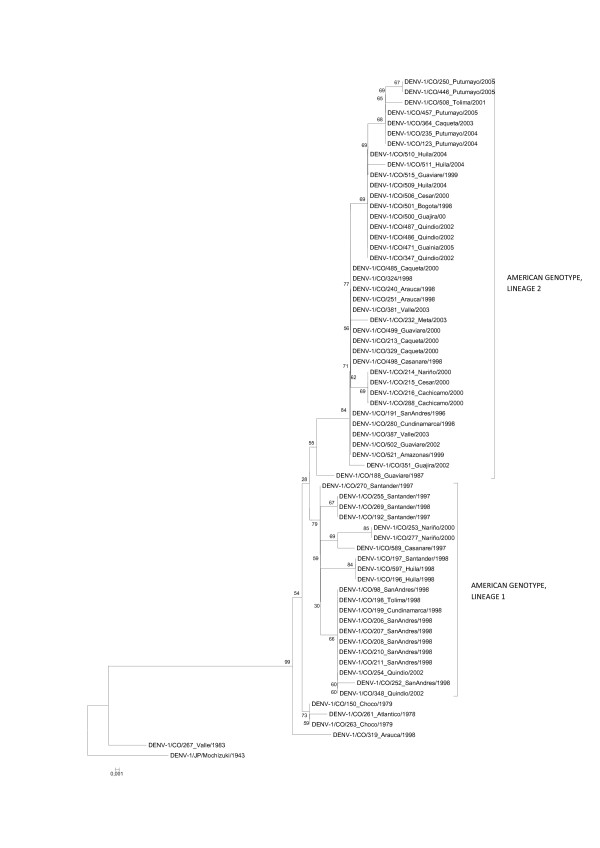
**Evolutionary relationships of DEN-1 Colombian isolates**. The evolutionary history was inferred using the Maximum Likelihood method. The optimal tree with the sum of branch length = 0.20353432 is shown. The confidence probability of each node was estimated using the bootstrap test (1000 replicates) and values over 50% are presented. The tree is drawn to scale, with branch lengths in the same units as those of the evolutionary distances used to infer the phylogenetic tree. The evolutionary distances were computed using the Maximum Likelihood method and are in the units of the number of base substitutions per site. Square brackets indicate putative lineages 1 and 2. Phylogenetic analyses were conducted in PAUP*

DENV-1/CO/261_Atlantico/1978, DENV-1/CO/263_Choco/1979 and DENV-1/CO/150_Choco/1979 were slightly distant from the remaining strains and appeared in close proximity to some Caribbean Island and other American isolates (Trinidad, French Guinea). Interestingly, the isolate DENV-1/CO/267_Valle/1983 appeared in a different clade, as the sister branch of Japan (DENV-1/JP/Mochizuki/1943), China (DENV-1/CN/GZ-80/1980), Ethiopia (DENV-1/DJ/Ethiopia/1998) and Cambodia (DENV-1/KH/1998) strains, which have been defined as lineages of the genotype I [26]. To allow a better resolution of the tree, we performed a phylogenetic reconstruction using only the Colombian isolates. Again, the oldest isolates were more divergent representing the first entrance of virus in Colombia. Although the genotype V was the only represented in the tree, two different lineages may be defined based on cladal distribution (figure 2).

### Molecular clock

**Figure 3:**
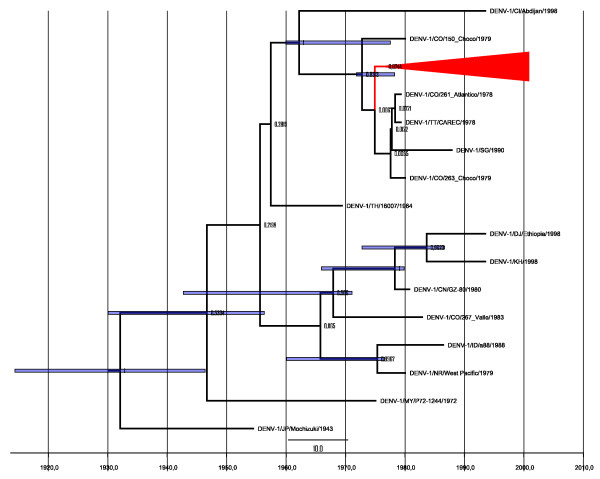
**Molecular Clock of DENV-1**. DENV-1 divergence time was estimated using year of isolation (scale used in the tree) as calibration points under the strict molecular clock model using GTR+Γ+I parameters. Posterior Probability (PP) values are indicated for each node, and the extent of the 95% highest posterior density (HPD) intervals for each divergence time is represented by the blue bars. Branches representing America genotype including Colombian isolates were collapsed (presented in red) for clearness purposes. Root of the tree was previously calculated by TRACE statistic interface of BEAST, and is placed in 1924 as the most recent ancestor

**Figure 4:**
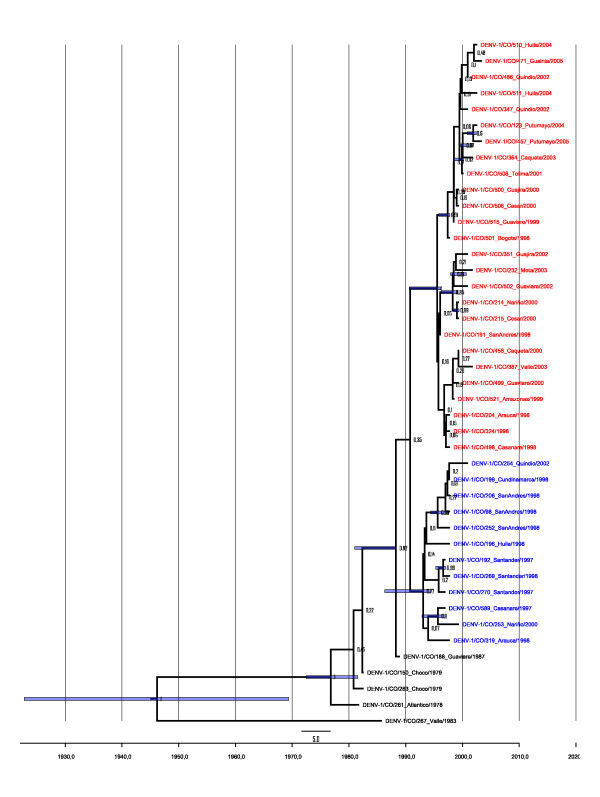
**Molecular Clock of DENV-1 Colombian Isolates**. Divergence time of DENV-1 Colombian isolates was estimated using year of isolation (scale used in the tree) as calibration points under the strict molecular clock model using GTR+Γ+I parameters. Posterior Probability (PP) values are indicated for each node, and the extent of the 95% highest posterior density (HPD) intervals for each divergence time is represented by the blue bars. Root of the tree was previously calculated by TRACE statistic interface of BEAST, and is placed in 1945 as the most recent ancestor. Taxa from the putative lineages 1 and 2 are shown in blue and red, respectively.

We used a Bayesian inference based on MCMC to reconstruct Colombian DENV 1 coalescent history. BEAST allowed the use of isolation year as calibrating point to estimate divergence time and then generated a posterior probability (PP) distribution of trees instead of a bootstrap value [41-44]. The resulting tree clearly placed the genotypes of DENV-1 already circulating globally before the first appearance of this serotype in the Americas between 1970 and 1980 (figure 3). According to the 95% highest posterior density (HPD) beneath the strict clock model, the estimated root for this phylogeny was 1929 and the substitution rate was 8.58 × 10^-4 ^substitutions per site, per year. To increase resolution, we use the strict molecular clock model to reconstruct Colombian isolates (figure 4). Under the assumption of a constant substitution rate, the estimated root indicates 1945 as the date of the more recent common ancestor. In addition, there was a split up around 1987 between DENV-1/CO/188_Guaviare/1987 isolates and the remaining strains (PP = 0,82). As the time goes by, we can see a sustained increase in number of isolates and a rapid spread of viruses, which included few changes among them as seen with the branch lengths. By the year 1992 (approximately), another remarkable partition event occurred to generate 2 well defined clades (PP = 0,77 and 1), evolving independently since the early 90's until recent time.

## Discussion

Emerging and re-emerging diseases have become a public health major concern in developing countries, where dengue is perhaps the most important vector-borne viral disease in terms of morbidity. In Colombia, DF and DHF had been associated to the four DENV serotypes with DENV-2 and DENV-1 predominating since 1971 after the re-appearance and spread of *Aedes (Stegomyia)aegipty *[6]. DENV-3 circulated for a short time in 1975 and then it was not detected until 2002 when re-introduction occurred probably from Venezuela [27]. DENV-4 has been detected sporadically every year since 1984, when it was involved in several DF cases.

The huge genetic diversity of DENV has been vastly documented, starting perhaps with the Rico-Hesse proposal of different "genotypes" comprising serotypes 1 and 2 [10], following by several studies and genotype definition of DENV-3 and DENV-4. In this way, five different genotypes has been previously defined for DENV-1 (genotypes I to V) suggesting a significant genetic variation. In fact, various lineages had been proposed based on time-spatial clustering and clade distribution [26,28,35-38]. In the present study, 74 Colombian DENV-1 sequences were analyzed to try to reconstruct the phylogenetic history of the virus in this country. Different genome regions have been used to infer DENV phylogeny including those with short fragments [10,27,28]. Here we employed a sequence from the carboxi terminal of the envelope (E) protein which has demonstrated to provide a useful phylogenetic signal to define genotype clustering [26]. It is important to note that the better resolution of evolutionary patterns should be obtained from complete genomes. However, it was not possible to obtain largest fragments from the oldest isolates, probably because of RNA degradation across the time. As expected, all strains were clustered with those from Brazil, Paraguay, Argentina, and different Caribbean Islands, corresponding to the formerly named genotype V (America/Africa), showing a well supported clade clearly separated from the others genotypes. Colombian strains DENV-1/CO/261_Atlantico/1978, DENV-1/CO/263_Choco/1979 and DENV-1/CO/150_Choco/1979, were separated from the remaining isolates and appeared closer to those from the Caribbean islands, which represent the entrance of serotype 1 into the Americas. It was reported for the first time in 1977 in Jamaica and rapidly spreading to the Antilles including Cuba, Antigua & Barbuda, Aruba, Bahamas, Barbados, Curaçao, Dominica, Grenada, Guadaloupe, Guyana, Haiti, Martinique, Montserrat, Puerto Rico, St. Kitts, St. Martin, St. Vincent and the Grenadines, Trinidad, Turks and Caicos, and the Virgin Islands [5]. In 1978, DENV-1 was implicated in large mainland outbreaks perhaps occurring at the same time in Colombia, Venezuela, Surinam, French Guyana, and eventually Centro America and Mexico. In Colombia, DENV-1 was isolated between 1977 and 1978, so the strain DENV-1/CO/261_Atlantico/1978 represents perhaps the first virus entrance to the country. It rapidly spread until the next isolation in Choco (DENV-1/CO/263_Choco/1979 and DENV-1/CO/150_Choco/1979) and then it fades away (or at less it was not reported) probably displaced by DENV-2 (maintaining DENV-1 in a silent low circulation) until 1985 when it established in different localities. It is important to note that even with the mobility between countries and increasing opportunity of viral introduction, only one DENV-1 genotype is circulating in America, different to DENV-2 and DENV-3 of which at least 2 genotypes has been detected (America/Asia genotypes and I/III genotypes respectively) suggesting perhaps, dissimilar patterns of viral spread and transmission between DENV genotypes and even different adaptation capacity.

Many researchers have categorized DENV in non official taxonomic levels beneath genotype, based specially in cladal distribution or geographical clustering. Circulation of these "lineages" has been particularly defined for DENV-1 in India, where at least 4 different lineages had been proposed (India-1 close to American strains, India-2 related to Singapore 1993 isolate, India-3 in south India and India-4 from Delhi and Gwalior) [26,28]. In our study, a remarkable cladogenesis event occurs around 1992 according to the molecular clock, generating two well supported clades corresponding to putative Colombian DENV-1 lineages. Despite the eco-epidemiology similarities between Colombia and neighbor countries were dengue is a major concern, lineages have not been previously demonstrated for DENV-1. In fact, according to ML phylogeny, most of the American strains (Argentina and Brazil) correspond to the lineage-1, leaving the lineage 2 restricted to Colombia. Although geographic distribution of these lineages is not clearly delimitated, it is evident that they are evolving independently and most likely in parallel at the same localities.

Despite the emergence and rapid diversification of DENV has been a matter of special concern, precise mechanisms of evolution remain unclear [45-50]. It is a fact that human RNA viruses including Influenza, HIV, Coronavirus, etc., have particularly increased mutation and evolution rates mostly because of the lack of proofreading activity of RNA-dependent RNA-polymerase [51,52]. Nevertheless, arthropod-borne viruses (Arboviruses) have demonstrated slower mutation rates comparing with those infecting directly human host, probably because of the trade-off effect occurring when the virus is obligated to adapt alternatively into the invertebrate vector and vertebrate host [51]. This resulting constrain has been experimentally assessed *in vivo *to Venezuelan Equine Encephalitis [52] and *in vitro *to DENV [51] demonstrating that fitness improves when virus specialize in a single cell line but decreases in virus undergoing alternative passages in different cells. In view of that, over all DENV mutation rates have been previously inferred, ranging from 4.55 × 10^-4 ^(DENV-1) to 9.01 × 10^-4 ^(DENV-3) [19]. In the present study, we found a mutation rate of 8.58 × 10^-4 ^substitutions per site, per year, suggesting faster evolution rates for Colombian strains, perhaps because of the high transmition occurrence especially in hyperendemic areas, where virus replicates in several human hosts, reducing the constraining effect occurred in the vector. However, this high mutation rate does not necessarily reflect a fitness advantage or a successful adaptation process. Actually, positive selection for DENV seems to be serotype/genotype dependent and even more, protein specific. In fact, envelope (E) protein apparently exhibits some adaptation evidence in DENV-3, DENV-4 and various DENV-2 genotypes, but not for DENV-1, strongly suggesting a purifying selection pressure, at least over this gene. Nevertheless, further studies have to be done to try to understand the adaptation process in DENV.

On the other hand, although mostly of Colombian strains belong to the genotype V, there is an isolate, DENV-1/CO/267_Valle/1983 placed into genotype I near to Asia, China and East Africa strains. The ML tree show this strain close to DENV-1/JP/Mochizuki/1943, a strain considered extinct. Since we do not have this virus as reference in our laboratory, we can discard cross contamination during the assay. Moreover, the presence of this virus could be explained based on the migration process occurred from Asia to America, officially starting to Colombia by 1929, and sustained until the mid XX century [53]. Thus, establishment of Asian colonies increased visitors and perhaps favored the entrance of viral strains. We can speculate that those viruses did not fit to the new environment and the adaptation events were constrained because of the selective pressures including different vectors and human immune response.

According to natural history of DENV, evolution events could bring new genetic variants and eventually increase the severity of disease. Although pathogenic markers remain unclear, hemorrhagic features on some Asian DENV-2 genotypes have been demonstrated and Asian derived DENV-3 genotypes associated to dengue fever and dengue hemorrhagic fever have been reported in Brazil [25]. Moreover, changes in clinical manifestation of disease (atypical dengue) such as viscerotropism or encephalitis may respond to the circulation of new DENV lineages with increased pathogenic potential. Consequently, epidemiological programs should include not just virological diagnosis but genotype surveillance too.

## Conclusion

This study shows in a defined time-scale, not just the first entrance of DENV 1 in Colombia, but also the viral evolution process in a highly endemic area. As a major conclusion, only one genotype of DENV 1 has been circulating since the first epidemic reports in the continental area. Nevertheless, two different lineages have been evolving fast since the earliest 90's according to molecular clock. As these evolution events may derive in a marked pathogenic potential, surveillance programs should include molecular methodologies. In fact, unusual presentation of disease currently reported by local health care institutions may be correlated to this evolution process. Further analyses by using at least complete E gene should be done to corroborate our results.

## Methods

### Virus strains

DENV-1 strains used in this study were obtained from the virus collection of the National Health Institute (INS, Virology Lab, Bogotá, Colombia), and comprise 74 isolates from outbreaks, epidemics and routine epidemiological surveillance. Clinical samples were collected between 1978 and 2007 from different localities all around the country, so they represent most viruses circulating in Colombia during the last 30 years (Table 1). All viral stocks were inoculated on C6/36 *Aedes albopictus *cells growing in Eagle's minimal essential medium (E-MEM) supplemented with 2% fetal calf serum (FSC). After 10 days of incubation at 28°C, monolayer was disrupted and supernatant was then recovered by centrifugation and stored at -80°C until use. The remained cells were washed with Phosfate Buffer Saline (PBS) and dripped on slides; after fixed in cold acetone, slides were incubated with monoclonal antibodies (anti-DENV-1 to anti-DENV-4, kindly donated by CDC, Puerto Rico) for one hour, washed with PBS and incubated again with a fluorescent conjugated antibody. Additionally, DENV-1 serotype confirmation was done by reverse transcription polymerase chain reaction (RT-PCR) using specific primers [54].

### Viral RNA extraction, RT-PCR and sequencing

Cell culture supernatants were used to extract viral RNA using QIAamp Viral RNA Minikit (Qiagen, Germany) following manufacturer's instructions. Briefly, 140 μl of each supernatant was placed into 560 μl of AVL buffer with 5.6 μl of carrier RNA and mixed with ethanol (96-100%) before passed through a column by centrifugation. After washing with buffers AW1 and AW2 RNA was finally eluted with 60 μl of AVE buffer and stored at -80°C until use. Five microliters from each RNA extraction were used as template in a one step RT-PCR reaction (Qiagen, One-Step RT-PCR kit) as previously described [55]. Primers used [DEN1S1871 (5'-TGGCTGAGACCCARCATGGNAC-3') and DEN1AS2622 (5'-CAATTCATTTGATATTTGYTTCCAC-3')] were designated to amplify 751 bp from de joining region E/NS1. Reactions were evaluated in 1% agarose gel stained with ethidum bromide and reactions observed as negative were then subjected to nested PCR as follow: 1 μl of initial RT-PCR product, 1 × buffer B (60 mM Tris-HCl pH 8.5, 2 mM MgCl_2_, 15 mM (NH_4_)_2_SO_4_), 40 pmol of each primer [DEN1S2133 (5'-GGAAAATGTTYGAAGCAACYGCCC-3'), DEN1AS2553 (5'-TCCTCCCATGCCTTCCCRATGG-3')] and 2.5 U of Taq DNA Polimerase (Invotrogene) in a final volume of 50 μl. PCR reactions were first denaturated at 94°C (2 minutes) and then subjected to 40 cycles of denaturation (94°C, 30 seconds), primer annealing (57°C, 4 minutes), primer extension (72°C, 30 seconds) and a final extension step at 72°C for 5 minutes. Nested PCR was evaluated in 1% agarose gel stained with ethidium bromide.

Amplified products (from RT-PCR or nested PCR) were purified using QIAquick PCR Purification Kit (QIAGEN, Germany) and then used as template for sequencing reactions using the ABI Prism Dye Terminator Cycle Sequencing Ready Reaction Kit (Applied Biosystems, Foster City, CA). Sequencing was carried out on both strands with 10 pmol of primers used for nested PCR, and the products were analyzed using an ABI model 377 automated sequencer (Applied Biosystems, USA). Overlapping sequences for each sample obtained from sense and antisense primers were combined to obtain a consensus sequence using the SeqMan module of Lasergene (DNASTAR Inc. Software, Madison, Wis.). A total of 224 bp [corresponding to carboxyl terminus of envelope (E) gene] from 74 new sequences were compared with 52 previously sequenced strains from all over the world, available in GenBank. Consensus sequences were aligned using the program CLUSTAL W included in MEGA package version 4.0 [35,36].

### Phylogenetic analyses

Phylogenetic trees were constructed with the Maximum Parsimony and Maximum Likelihood (ML) methods incorporated in the PAUP* 4.0 program [56]. Phylogenetic analyses were performed by using the best model of nucleotide substitution based on Modeltest [57] (analyses are available upon request). Statistical significance of tree topology was assessed with a bootstrap with 1000 replicates. Obtained trees were visualized using the Tree View Program [58].

### Substitution rates and molecular clock

In addition, estimated rate of evolutionary change (nucleotide substitutions per site per year) and tree root age was obtained with the program BEAST (Bayesian Evolutionary Analysis by Sampling Trees)[41], which uses Bayesian Markov Chain Montecarlo (MCMC) algorithms combined with the chosen model and prior knowledge of sequence data to infer the posterior probability distribution of phylogenies [41-44]. We analyze the data using the year of isolation as calibration points to estimate divergence time in years. In order to avoid duplicates, sequences identical to other on the dataset were removed. Rate variation among branches was inferred under the strict molecular clock model, whereas substitution rate among sites was calculated with the General Time-Reversible model (GTR) combined with the gamma parameter and proportion of invariant sites (GTR+Γ+I ) model. MCMC was run for 10,000,000 steps and sampled every 500 steps and the 10,000 first steps of each run were discarded. BEAST format files were obtained in the provided BEAUti graphical interface and the generated trees were visualized with the FigTree 1.2.2. program. Finally, statistical analysis was carried out in the Tracer package [41].

## Competing interests

The authors declare that they have no competing interests.

## Authors' contributions

JAMR contributed to the experimental design, carried out the experiments and phylogenetic and molecular clock analysis, and wrote the manuscript. JAUC contributed to the experimental design, carried out the experiments and provided a critical review of the manuscript. CDC participated in the experimental design, contributed to the interpretation of data and the critical review of the manuscript. GJRB contributed to the experimental design and provided a critical review of the manuscript. JAS contributed with phylogenetic and molecular clock analysis and BEAST running and provided a critical review of the manuscript. ATM conceived the study, its experimental design and provided a critical review of the manuscript. JCGG conceived the study, participated in its design and coordination and provide a final review of the manuscript. All authors read and approved the final version of the manuscript.
